# Evolving treatment implementation among HIV–infected pregnant women and their partners: results from a national surveillance study in Italy, 2001–2015

**DOI:** 10.7189/jogh.07.010407

**Published:** 2017-06

**Authors:** Marco Floridia, Valentina Frisina, Marina Ravizza, Anna Maria Marconi, Carmela Pinnetti, Irene Cetin, Matilde Sansone, Atim Molinari, Francesca Cervi, Alessandra Meloni, Kety Luzi, Giulia Masuelli, Enrica Tamburrini, The Italian Group on Surveillance on Antiretroviral Treatment in Pregnancy

**Affiliations:** 1 National Center for Global Health, Istituto Superiore di Sanità, Rome, Italy; 2 Department of Obstetrics and Neonatology, Città della Salute e della Scienza Hospital, and Universiy of Turin, Turin, Italy; 3 Department of Obstetrics and Gynaecology, DMSD San Paolo Hospital Medical School, University of Milan, Milan, Italy; 4 INMI Lazzaro Spallanzani, Rome, Italy; 5 Department of Obstetrics and Gynaecology, Luigi Sacco Hospital and University of Milan, Milan, Italy; 6 Department of Neurosciences, Reproductive and Dentistry Science, University Federico II, Naples, Naples Italy; 7 Department of Infectious Diseases and Hepatology, Azienda Ospedaliera di Parma, Parma, Italy; 8 Department of Medical and Surgical Sciences, Policlinico Sant’Orsola–Malpighi and University of Bologna, Bologna, Italy; 9 Division of Gynaecology and Obstetrics, S. Giovanni di Dio Hospital and University of Cagliari, Cagliari, Italy; 10 Department of Infectious Diseases, Misericordia e Dolce Hospital, Prato, Italy; 11 Department of Infectious Diseases, Catholic University, Rome, Italy

## Abstract

**Background:**

The current global and national indications for antiretroviral treatment (ART, usually triple combination therapy) in adolescent and adults, including pregnant women, recommend early ART before immunologic decline, pre–exposure chemoprophylaxis (PrEP), and treatment of HIV–negative partners in serodiscordant couples. There is limited information on the implementation of these recommendations among pregnant women with HIV and their partners.

**Methods:**

The present analysis was performed in 2016, using data from clinical records of pregnant women with HIV, followed between 2001 and 2015 at hospital or university clinics within a large, nationally representative Italian cohort study. The study period was divided in three intervals of five years each (2001–2005, 2006–2010, 2011–2015), and the analysis evaluated temporal trends in rates of HIV diagnosis in pregnancy, maternal antiretroviral treatment at conception, prevalence of HIV infection among partners of pregnant women with HIV, and proportion of seronegative and seropositive male partners receiving antiretroviral treatment.

**Results:**

The analysis included 2755 pregnancies in women with HIV. During the three time intervals considered the rate of HIV diagnosis in pregnancy (overall 23.3%), and the distribution of HIV status among male partners (overall 48.7% HIV–negative, 28.6% HIV–positive and 22.8% unknown) remained substantially unchanged. Significant increases were observed in the proportion of women with HIV diagnosed before pregnancy who were on antiretroviral treatment at conception (from 62.0% in 2001–2005 to 81.3% in 2011–2015, *P* < 0.001), and in the proportion of HIV–positive partners on antiretroviral treatment (from 73.3% in 2001–2005 to 95.8% in 2011–2015, *P* = 0.002). Antiretroviral treatment was administered in 99.1% of the pregnancies that did not end early because of miscarriage, termination, or intrauterine death, and in 75.3% of those not ending in a live birth. No implementation of antiretroviral treatment was introduced among male HIV–negative partners.

**Conclusions:**

The results suggest good implementation of antiretroviral treatment among HIV–positive women and their HIV–positive partners, but no implementation, even in recent years, of Pre–Exposure Prophylaxis (PrEP) among uninfected male partners. Further studies should assess the determinants of this occurrence and clarify the attitudes and the potential barriers to PrEP use.

The recent global HIV guidelines expanded the use of antiretroviral treatment (ART) to include early ART before immunologic decline, pre–exposure chemoprophylaxis (PrEP), and treatment of HIV–negative partners in serodiscordant couples [1–3]. All these situations apply to women with HIV who are planning a pregnancy or are already pregnant, and to their partners. An effective implementation of these measures is necessarily dependent on knowledge of HIV status in both partners, and a good implementation of HIV diagnosis is necessary to ensure effectiveness in the “cascade” process that link different steps of diagnosis and care of HIV (testing, implementation of treatment, adherence with treatment, effective suppression of viral load) [4]. If both the woman and the partner are infected, both should be on treatment according to the expanded indications of the new guidelines, irrespective of the level of immunological deterioration. Treatment is however also now recommended, although less stringently, for the HIV–uninfected male partners of HIV–positive women. In this context, it is important to quantify to what extent the new guidelines on treatment of HIV positive pregnant women and their HIV positive partners, or PrEP among HIV–negative partners in serodiscordant couples is implemented. In order to quantify this, we used data from a large national cohort of pregnant women with HIV to explore temporal trends in the proportion of HIV diagnoses that occurred in pregnancy, maternal antiretroviral treatment at conception, prevalence of HIV infection among partners of HIV positive pregnant women, and proportion of seronegative and seropositive male partners receiving antiretroviral treatment.

## METHODS

Data from the Italian National Program on Surveillance of Antiretroviral Treatment in Pregnancy were used [5]. This is an ongoing observational study established in Italy in 2001, which covers, based on available prevalence data [6], roughly 30–40% of live births from women with HIV in Italy. Site participation is voluntary, and at present is based on more than 30 clinicians reporting from Obstetrics, Infectious Diseases, and Paediatrics departments across the country. Only HIV–positive pregnant women are included, and treatment of HIV infection is decided by the treating physician, usually according to national guidelines. Laboratory and clinical data are collected from hospital records, following the women’s consent based on a patient information sheet approved by the competent Ethics Committee. Information and measurements are collected at routine pregnancy visits, at delivery, and during a follow–up of mothers and newborns for up to 18 months. Timing of HIV diagnosis is calculated using the date difference between HIV diagnosis and last menstrual period. Gestational age at birth is determined on the basis of the last menstrual period, ultrasound biometry, or both. Information on HIV status of the partners is based on women’s report. Women provide consent based on a patient information sheet approved by the competent Ethics Committee (deliberation 578, September 28, 2001, I.N.M.I. Lazzaro Spallanzani Ethics Committee, Rome). For the current analysis, all pregnancies with available date of HIV diagnosis were considered eligible. The study period (2001–2015) was divided in three intervals of five years each (2001–2005, 2006–2010, 2011–2015), and temporal trends were analyzed using the chi–square test for trend. *P* values <0.05 were considered significant. All statistical analyses were performed with the SPSS software, version 22 (IBM Corp, released 2013, Armonk, NY, USA).

## RESULTS

As of 19 August 2016, 2755 pregnancies in HIV–infected women had available information and were included in the analyses. The temporal trends are summarized in **Table 1**. Across the three time periods studied, the rate of HIV diagnosis in pregnancy remained stable (overall: 643/2755, 23.3%), with no significant changes over time (*P* = 0.908). Conversely, the proportion of women with HIV diagnosed before pregnancy who were on ART at conception increased significantly from 62.0% in 2001–2005 (557/899), to 81.3% (312/384) in 2011–2015 (*P* < 0.001). Subsequent ART coverage in pregnancy was roughly universal, involving 99.1% (2306/2326) of the pregnancies that did not end early because of miscarriage, termination, or intrauterine death (proportion on ART in this group with no live births: 75.3%, 235/312).

**Table 1:** Temporal trends in HIV and treatment status of mothers and partners (2001–2015)

	2001–2015	2001–2005	2006–2010	2011–2015	*P* value*
**Diagnosis of HIV in current pregnancy (%)**	23.3 (643/2755)	22.9 (268/1172)	24.2 (261/1079)	22.6 (114/504)	0.908
**Mothers on treatment at conception (%):**					
Overall	51.8 (1414/2728)	47.8 (557/1166)	51.0 (545/1068)	63.2 (312/494)	<0.001
HIV known before pregnancy	67.6 (1414/2093)	62.0 (557/899)	67.3 (545/810)	81.3 (312/384)	<0.001
**HIV status of the partner† (%):**					
HIV–positive	28.6 (593/2077)	29.4 (262/892)	28.3 (228/807)	27.2 (103/378)	0.423
HIV–negative	48.7 (1011/2077)	50.4 (450/892)	46.0 (371/807)	50.3 (190/378)	0.562
Unknown	22.8 (473/2077)	20.2 (180/892)	25.8 (208/807)	22.5 (85/378)	0.120
HIV–positive partners on treatment† (%)	77.0 (291/378)	73.3 (121/165)	71.8 (102/142)	95.8 (68/71)	0.002
**Guidelines recommendations for treatment in pregnancy**		Recommended (usually mono–or dual therapy)	Recommended (combination therapy)	Recommended (combination therapy)	
**Guidelines recommendations for treatment in all HIV–infected individuals**		<200–350 CD4 or symptomatic disease	<350–500 CD4 or symptomatic disease	Progressive shift to any CD4 level	
**Guidelines recommendations for treatment in HIV–negative partners**		Not recommended	Not recommended	PrEP (since 2012)	

*χ^2^ for trend.

†Women with HIV infection diagnosed before pregnancy only. No HIV–negative partner was receiving anti–HIV treatment across the entire period of observation.

The distribution of HIV status of male partners remained substantially unchanged during the entire period (overall: 48.7% HIV–negative, 28.6% HIV–positive and 22.8% unknown). None of the HIV–negative partners received antiretroviral treatment during the entire time of observation, while the proportion of HIV–positive partners receiving treatment increased significantly from 73.3% in 2001–2005 to 95.8% in 2011–2015 (*P* = 0.002, **Table 1**).

## CONCLUSION

This analysis provided information on several aspects of the cascade of HIV diagnosis and treatment among pregnant women with HIV and their partners that can be relevant for health care providers. At first, we showed a stable rate of HIV diagnosis during pregnancy in the last 15 years. Although this rate (23%) is similar or even lower compared to other national and international studies [7,8], this suggests no improvement in the proportion of cases in which HIV infection was already known before pregnancy, a condition that represents the target for an optimal management of pregnancy. Similarly, no temporal changes were observed in the distribution of HIV status of the partners, with roughly half of the pregnancies occurring in a context of HIV–serodiscordant couples (with an HIV–uninfected male partner), and no less than 20% of cases (overlapping the proportion of maternal HIV diagnosis in pregnancy) characterized by an unknown HIV status of the partner, suggesting frequent occurrence of no HIV testing for both members of the couple before pregnancy. These findings indicate that there is still the need to improve the rate of HIV testing among people of childbearing age through information campaigns and facilitated access to testing and prenatal counselling services. Such services should be targeted not only to women who are planning pregnancy or who present in early pregnancy, but also to their partners. This not only would improve management of pregnancy but also clinical HIV outcomes.

The analysis of temporal trends showed significant improvements in treatment coverage in the population with HIV already known before pregnancy. In 2011–2015, more than 80% of the women diagnosed before pregnancy were on treatment at conception, and almost all their HIV–positive partners (95.8%) were receiving ART. These figures indicate good implementation of the expanded indications to ART among people with HIV in Italy, confirming a good response to the recent guidelines that have progressively expanded the indication to treatment to any person with HIV, irrespective of the level of immune deterioration [1,2,9,10]. A wide effort is ongoing worldwide to ensure equal access to antiretroviral treatment to all infected people, with the aim to obtain the 90–90–90 target (90% diagnosed, 90% treated and 90% virally suppressed), and ultimately end the HIV/AIDS epidemics by 2030. Encouraging results have been achieved, even in countries with lower resources, with more than 17 millions of people on antiretroviral treatment in 2016, although large disparities exist between countries [4]. Current challenges in this pathway involve reaching and diagnosing the millions of people who do not know that they have HIV, and effectively retaining in care those who start ART, in order to achieve a prolonged viral suppression [11].

Even more challenging may be providing treatment as prophylaxis to HIV–uninfected partners. In our study, none of the HIV–uninfected males in the serodiscordant couples, even in recent years, received antiretroviral treatment as a potential prophylaxis against HIV transmission. The absence of any PrEP among uninfected males indicates no implementation of the recently recommended use of antiretroviral treatment as a preventive measure in uninfected partners. We are unable to define the possible basis for this occurrence, that may include preference for barrier methods compared to antiretroviral treatment as preventive measures against HIV transmission in sexually active serodiscordant couples, with use of self–insemination techniques (in which the woman inserts semen into the vagina herself, without medical intervention) when attempting to conceive. The implementation and efficacy of guidelines may be affected by several factors that influence the attitudes of both health care providers and people with HIV. The efficacy of PreP, in particular, is conditioned by adherence, perceived level of HIV risk, and access and availability of health services [12]. All these factors may show important differences among male and female heterosexuals, sex workers, men who have sex with men, and injection drug users [13,14]. Several studies have shown the difficulty to enrol partners of HIV–infected pregnant women into programs of HIV testing and care [15,16]. Our results indicate the need for further studies that investigate the potential barriers to PrEP implementation in uninfected partners of HIV–infected women, including the specific attitudes and expectations on PrEP use among prescribing physicians and people with HIV.
